# Sintilimab-Induced Myocarditis in a Patient with Gastric Cancer: A Case Report and Literature Review

**DOI:** 10.3390/jcdd10100422

**Published:** 2023-10-09

**Authors:** Xin Liu, Ziyue Zeng, Jianlei Cao, Xianqing Li, Muheremu Muhetaer, Zhili Jin, Huanhuan Cai, Zhibing Lu

**Affiliations:** 1 Department of Cardiology, Zhongnan Hospital, Wuhan University, No. 169 Donghu Road, Wuchang District, Wuhan 430071, China; 2021203030016@whu.edu.cn (X.L.); zengziyue001@163.com (Z.Z.); cjlzn14@whu.edu.cn (J.C.); lixianqing@whu.edu.cn (X.L.); 2021283030105@whu.edu.cn (M.M.); jinzhili@whu.edu.cn (Z.J.); 2 Institute of Myocardial Injury and Repair, Wuhan University, Wuhan 430071, China

**Keywords:** gastric cancer, immune checkpoint inhibitor, programmed cell death receptor-1, sintilimab, myocarditis

## Abstract

Immune checkpoint inhibitors (ICIs) have emerged as a powerful and efficacious therapeutic approach for many cancer patients. Sintilimab is a fully human IgG4 monoclonal antibody that binds with programmed cell death receptor-1 (PD-1) to block its interaction with ligands, thereby enhancing the antitumor effects of T cells. However, ICIs may induce immune-related adverse events (irAEs) in various systems and organs, with fulminant myocarditis being the most severe one. We report the case of a 45-year-old female with gastric cancer who developed chest pain two weeks after chemotherapy with sintilimab; she was diagnosed with immune-associated fulminant myocarditis and experienced an Adams–Stokes syndrome attack in the hospital. Eventually, she was discharged after being treated with methylprednisolone, immunoglobulin, and an IABP.

## 1. Introduction

Myocarditis is an inflammatory condition that affects the heart and can be triggered by various factors such as infections, immune system activation, or exposure to certain medications [1]. Notable causes of myocarditis include viruses like influenza and coronaviruses, autoimmune disorders such as systemic lupus erythematosus, certain medications like immune checkpoint inhibitors, as well as vaccinations like smallpox and mRNA vaccines for COVID-19 [2].

Numerous medications, especially chemotherapeutic agents and specific natural compounds [3], pose a risk to cardiomyocytes, as evidenced by the increasing incidence of fulminant myocarditis (FM) cases associated with immune checkpoint inhibitors [4]. While these inhibitors have shown remarkable efficacy in treating advanced malignancies, they also trigger the development of auto-cytotoxic immune cells that target the heart muscle. This leads to an influx of immune cells, including CD3^+^ CD8^+^ T lymphocytes, macrophages, and neutrophils, into the cardiac tissue [5,6]. The suppression of immune regulation contributes to immune-related adverse events, which have been documented across various systems, such as the nervous, endocrine, respiratory, gastrointestinal, and renal systems, upon their initial regulatory approval in 2014. Checkpoint inhibitors exhibit a notably high prevalence of cardiac adverse effects, potentially exceeding 25% [7]. While the incidence of FM resulting from checkpoint inhibitors remains below 1%, which is considerably lower than other adverse events, its fatality rate is alarmingly high, ranging from 40% to 70% [8,9,10].

In this report, we present a case of a 45-year-old female with advanced gastric cancer who experienced fulminant myocarditis and Adams–Stokes syndrome. Effective treatment involved methylprednisolone, immunoglobulin, and intra-aortic balloon pump (IABP) therapy. This study aims to share insights into diagnosing and managing ICI-induced myocarditis.

## 2. Case Presentation

A 45-year-old female with a history of advanced gastric cancer was admitted to our hospital with chest pain and palpitations after completing a round of chemotherapy that included sintilimab and tegafur, gimeracil, and oteracil potassium capsule (S-1) plus oxaliplatin (SOX regimen). The patient was diagnosed with advanced gastric cancer in July 2022. Following a thorough evaluation of her condition, she began tegafur plus oxaliplatin chemotherapy in combination with sintilimab (200 mg). The patient developed chest discomfort and palpitations 4 weeks following sintilimab administration. After experiencing recurring chest discomfort associated with changes in vocal quality, diminished muscular strength, and impaired cognitive function over a period of two weeks, the patient presented to our medical institution on 10 September. She had no history of hypertension, diabetes, dyslipidemia, smoking, or drinking. At the time of admission, the vital signs of the patient were stable and her physical examination was normal. The muscle strength of the extremities was normal and there was no edema in the lower limbs.

**Figure 1:**
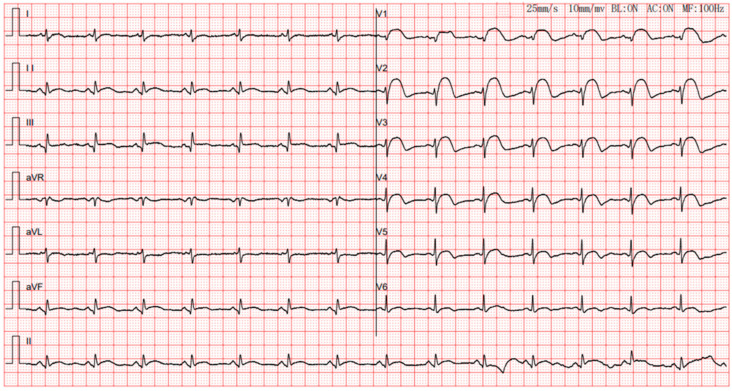
ECG of the patient upon admission. Electrocardiogram shows sinus rhythm and inferior wall, septal wall, and anterior wall resembling myocardial infarction.

**Figure 2:**
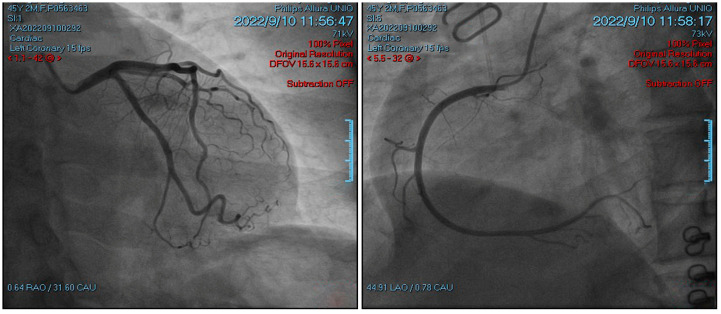
Coronary angiography upon admission. The left figure is the left coronary artery, and the right figure is the right coronary artery.

**Figure 3:**
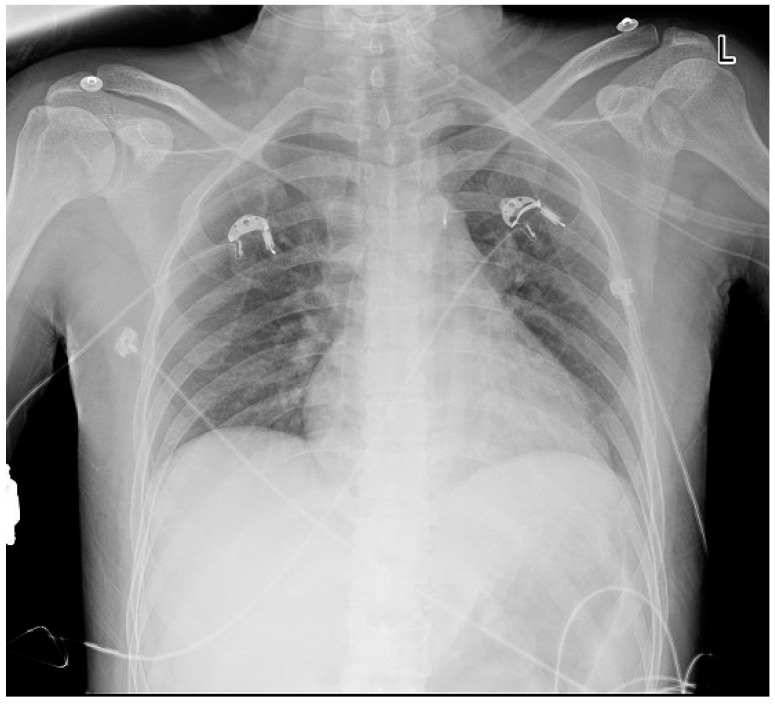
Chest X-ray shows significant cardiomegaly upon admission. The heart-to-chest ratio is 0.6.

The patient showed markedly increased highly sensitive troponin I (HSTNI) at 5574 pg/mL (reference range: 0–26.2), N-terminal pro-B-type natriuretic peptide (NT-proBNP) at 111.9 pg/mL (reference range: <100), creatine kinase isoenzyme MB (CK-MB) at 96.7 ng/ul (reference range: 0–6.6), D-dimer at 512 ng/mL (reference range: 80–500), PCO_2_ at 34.8 mmHg (reference range: 35–45), and PO_2_ at 148 mmHg (reference range: 83–108). Laboratory exams of electrolyte and renal functions were normal. ECG demonstrated ST-segment elevation in multiple leads (Figure 1). Echocardiography indicated dilated right ventricle (RV, 4.5 cm) and right atrium (RA, 4.6 cm), tricuspid regurgitation, but a preserved ejection fraction of 68%. Emergency coronary angiography revealed normal epicardial coronary arteries (Figure 2). A chest radiograph showed an enlarged heart shadow and a heart-to-chest ratio of 0.6 (Figure 3). After considering the patient’s history and examination results, ICI-mediated fulminant myocarditis was highly suspected. Based on the information provided, immunosuppressive medication with corticosteroids (methylprednisolone at 120 mg) was administered. Simultaneously, symptomatic, supportive therapies, such as atorvastatin, clopidogrel bisulfate, pantoprazole sodium, and insulin, as well as pain relief medications, were initiated.

**Figure 4:**
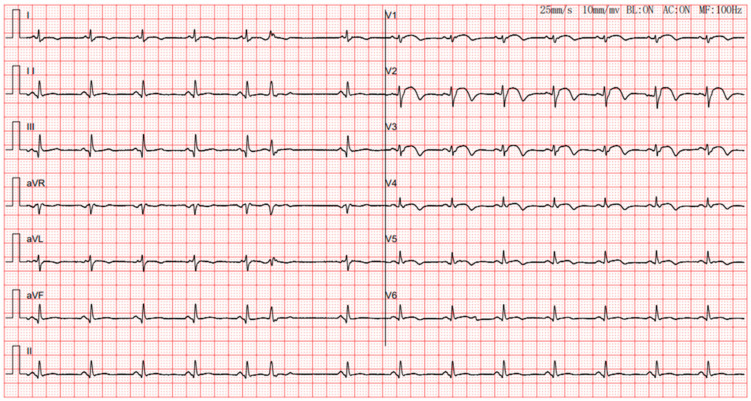
ECG during the course of comprehensive treatment. Electrocardiogram shows sinus rhythm and inferior wall, septal wall, and anterior wall resembling myocardial infarction.

The patient appeared to stabilize under steroid treatment. However, on September 11, the patient suffered from abrupt impairment of consciousness and seizures, followed by sudden respiratory arrest. ECG monitoring showed ventricular tachycardia and ventricular fibrillation. Continuous chest compressions were promptly instituted, and symptomatic epinephrine therapy was delivered to accelerate the heart. Her heart rate recovered after defibrillation. Simultaneously, an IABP was inserted. The patient’s heart rate ranged between 70 and 90 beats per minute after successful cardiopulmonary resuscitation, with stable blood pressure. The patient received intravenous methylprednisolone (120 mg) and immunoglobulin (20 g) for seven days. She showed markedly increased fasting blood glucose at 8.29 mmol/L (reference range: 3.9–6.1). Aspartate aminotransferase (AST) and alanine aminotransferase (ALT) levels were 183 U/L (reference range:13–35) and 98 U/L (reference range:7–45), respectively, on September 11. On September 12, ECG showed the elevated ST segment from the limb leads had fallen back, but ST-segment elevation persisted across the chest leads (Figure 4).

**Figure 5:**
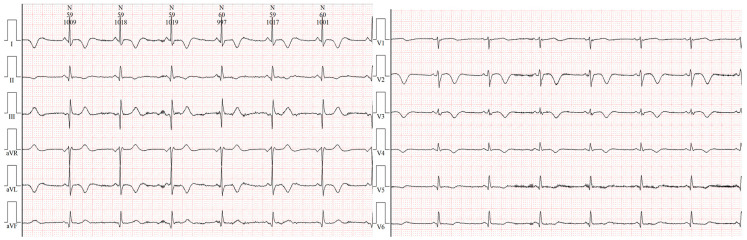
Holter monitor after comprehensive treatment. Holter monitor shows that elevated ST segment has decreased, indicating sinus rhythm and T wave inversion in some leads.

**Figure 6:**
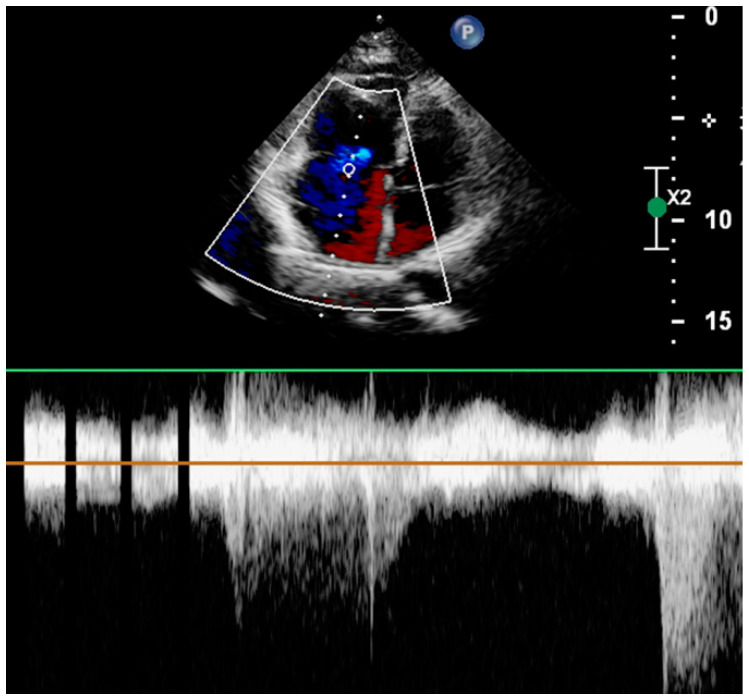
Electrocardiogram before discharge. Electrocardiogram shows right atrium and right ventricle have returned to normal size. There is mild tricuspid valve insufficiency.

The patient’s symptoms improved over time, and her physical parameters stabilized. HSTNI and myocardial enzyme indices in laboratory tests continued to decline. On the 7th day after admission, her Holter monitor showed sinus rhythm, a mean heart rate of 61 bpm, and no malignant arrhythmia (Figure 5). On 17 September, the IABP was removed. Her glucocorticoid medication dose was gradually lowered and adjusted to methylprednisolone at 100 mg/d. On September 20, her echocardiogram revealed that the RA (3.7 cm) and RV (3.6 cm) size values were within the normal reference range, and the ejection fraction was about 65% (Figure 6). After 11 days of hospitalization, the patient was discharged. She was given oral prednisone tablets, with gradual dose reductions and regular check-ups.

## 3. Discussion

In our report, we reported a case of gastric cancer treated with sintilimab and SOX regimen, which resulted in myocarditis. Adams–Stokes syndrome caused by ICI-induced myocarditis is uncommon in previous case reports. The combination of sintilimab and the SOX regimen caused fulminant myocarditis in this patient, resulting in seizures, impaired consciousness, and malignant arrhythmia, which finally resulted in an Adams–Stokes syndrome episode. Adams–Stokes syndrome is characterized by abrupt, transient loss of consciousness due to a rapid drop in cardiac output caused by sudden changes in the heart’s beating mechanism [11]. DeBoer has defined it as “every disturbance of the action of the heart that begins and ends abruptly and causes such interruption of the circulation that more or less complete cerebral ischemia results.” Clinically, there may be all grades of disturbances of consciousness ranging from a mild lightheadedness or dizziness to complete unconsciousness with convulsions [12].

Immune checkpoint inhibitors have revolutionized cancer treatment, with regulatory approval for 20 different cancer types [13]. The use of monoclonal antibodies to block immune regulators, like CTLA-4, PD-1, and PD-L1, can enhance T cell activity against cancer cells by preventing immune checkpoints from interfering. However, ICIs can also disrupt immune balance, triggering autoreactive T cells and various immune-related adverse events (irAEs), such as dermal, endocrine, and digestive toxicities. The reported incidence of irAEs varies depending on the targeted molecule, but they are encountered in around 60% to 90% of patients treated with ICIs, with the skin, digestive system, and liver being the most often infected tissues. On average, toxicities appear between 2 and 16 weeks after therapy begins. Although rare, cardiotoxicity induced by ICIs, including myocarditis, pericarditis, arrhythmias, myocardial ischemia, and valvular lesions, is serious and potentially fatal, with an incidence of 0.09–2.4% and a case fatality rate of 27–60% [14,15]. Major adverse cardiac events (MACEs), such as stroke, myocardial infarction, cardiovascular mortality, cardiogenic shock, cardiac arrest, and hospitalization for severe heart failure, occur in 46% of patients [16].

ICIs are monoclonal antibodies that precisely target and inhibit the action of immunological checkpoints [17]. CTLA-4 and PD-1 are the immunological checkpoints that ICIs effectively target. The immune checkpoints PD-1/PD-L1 and CTLA-4 are two distinct targets [18]. PD-1 is an immune checkpoint component that transmits inhibitory messages to T cells to reduce antitumor responses. It works with its ligand (mainly PD-L1) to suppress T cell activation. PD-1 inhibition is accountable for T cell function recovery, establishing the groundwork for anti-PD-1 treatment in tumor. T cell death is induced by PD-1 binding to PD-L1, resulting in T cell depletion and immunological evasion. PD-1/PD-L1 antibodies interrupt the interaction of PD-1 and PD-L1, resulting in T lymphocyte stimulation and multiplication. PD-1 is a negative co-stimulatory immunological molecule present on the surface of T cells, B cells, and bone marrow cells as a monomer. PD-L1 is a PD-1 ligand expressed in antigen-presenting cells and various kinds of carcinoma cells. It binds to PD-1 to trigger immunosuppressive signaling pathways, resulting in T cell inactivation and tumor immune evasion [19,20,21]. As a result, PD-1/PD-L1 inhibitors can boost the immune system’s reaction to malignancies by suppressing PD-1/PD-L1 interactions. In the treatment of advanced gastric cancer, PD-1/PD-L1 inhibitors have demonstrated great effectiveness in clinical trials [22].

In addition to ICI-induced myocarditis, we compared morbidity and mortality of other myocarditis types. Viral myocarditis, an infective cardiomyopathy triggered by viral infection, affects 10–22 individuals per 100,000, primarily young and middle-aged, and contributes to 12% of unexpected fatalities under the age of 40 [23,24]. Various viruses, including enteroviruses, adenoviruses, human parvoviruses, and human herpesviruses, can induce viral myocarditis [24,25]. Coxsackievirus B3 (CVB3), an enterovirus, is the most prevalent pathogen, infecting around 25% of cases. Although influenza is often a self-limiting respiratory infection, serious cardiac consequences, such as pericarditis resulting in tamponade and fulminant myocarditis causing shock, can occur. The fatality rate is 14.7%, with the majority of deaths occurring within 2 days of presentation due to cardiogenic shock and/or tamponade, demonstrating the aggressive character of this illness [26]. COVID-19, caused by SARS-CoV-2, presents cardiac implications in 20–30% of cases, which are linked to adverse outcomes and lead to higher in-hospital mortality in COVID-19 patients with myocarditis compared to patients with myocarditis alone (30.7% vs. 6.4%, with odds ratio of 4.8, 95% CI 3.7–6.3, P=0.001) [27]. ICI-induced and coronavirus disease 2019 (COVID-19) vaccine-induced myocarditis may have comparable pathways due to immune system overactivation. COVID-19 vaccine-induced myocarditis has emerged as a side effect of mRNA vaccines during the vast vaccination programs created in response to the COVID-19 pandemic, with an estimated incidence of 2.13 cases per 100,000 people [28]. The course of COVID-19 vaccine-induced myocarditis appears to be modest, with most patients fully recovering [29,30].

The Drug Reaction with Eosinophilia and Systemic Symptoms (DRESS) syndrome is a rare, potentially lethal drug reaction. It was previously called “drug-induced hypersensitivity syndrome” (DIHS) and “drug-induced delayed multiorgan hypersensitivity syndrome” (DIDMOHS). Dyspnea and chest discomfort are common symptoms in DRESS syndrome with cardiac involvement, which are particularly linked to minocycline and allopurinol, and are associated with a high mortality rate of 45.2% [31].

Diagnosing myocarditis, especially in ICI-treated patients, is challenging. A comprehensive approach involving biomarkers, cardiac imaging, and biopsy is recommended to rule out other causes, such as acute coronary syndrome [32]. Myocardial infiltration by T cells (CD4+ and CD8+), macrophages, and myocyte death are histological features of ICI-associated myocarditis, aligning with the Dallas criteria [33]. Cardiac MRI aids diagnosis [34,35]. Clinical manifestations of ICI-associated myocarditis vary, including early onset, arrhythmias, skeletal myositis, and myasthenia gravis, with a notable mortality rate [36]. While cardiac damage presents with elevated troponin levels, almost half of the patients show no systolic dysfunction, yet cardiac arrhythmias are widespread [16,37]. The “gold standard” for diagnosis of general myocarditis is histopathological evidence on endomyocardial biopsy or autopsy, although false negatives may occur from sampling error [38,39]. However, because of its invasive character, the danger of heart perforation, and the localized nature of biopsy samples, this test is not used as a first-line diagnostic tool, despite being considered the “gold standard.” Histological examination of a biopsy taken from an afflicted area may reveal inflammatory infiltrates (typically T-cell-predominant lymphocytic infiltrates) in the myocardium. Immunostains for cell-specific markers, including T lymphocytes (CD3), macrophages (CD68), or human leukocyte antigens, may improve the test’s sensitivity [40].

Among all kinds of ICI-induced cardiotoxicity, myocarditis has the greatest fatality rate. However, these side effects are frequently treated rapidly with steroids [41]. Immunosuppressive therapy, along with respiratory and circulatory support, is a mainstay of treatment [42]. Zhang et al. concluded in a retrospective investigation of 126 clinical cases that a high first dose (intravenous methylprednisolone at 1000 mg/d) and early corticosteroid administration (24 h) were linked with improved cardiac prognosis in ICI-associated myocarditis [43]. The treatment typically starts with intravenous methylprednisolone at 1000 mg daily for 3 days, followed by oral prednisone at 1 mg/kg/day with a slow taper over several weeks [44]. There are insufficient data regarding the most appropriate duration for corticosteroid medication, and it should be determined on an individual basis. In general, corticosteroids should be reduced gradually (during at least 4–6 weeks) and only when symptoms have been resolved, LVEF has been normalized, or arrhythmias have been stabilized [45]. Additional immunosuppressive strategies may be necessary for glucocorticoid-resistant cases. Supportive therapy, guided management, and proper medication, including beta blockers and renin–angiotensin II inhibitors, are recommended for heart failure. Intervention procedures, such as pericardiocentesis and pacemaker insertion, may be needed for specific cases. Impella support has shown effectiveness in myocarditis-induced cardiogenic shock [46].

## 4. Conclusions

In summary, oncologists should be cautious about ICI-induced myocarditis, especially when ICIs are administered in combination with chemotherapy. Before providing ICIs, patients’ cardiovascular status must be evaluated. This case suggests that timely identification and rapid implementation, including stopping immune checkpoint inhibitors and starting adequate steroid therapy and IABP implantation, may decrease ICI-related myocarditis and cardiogenic shock, thus lowering the risks of mortality and morbidity while improving prognosis.

## Data Availability

Not applicable.
